# Multi-Category Gesture Recognition Modeling Based on sEMG and IMU Signals

**DOI:** 10.3390/s22155855

**Published:** 2022-08-05

**Authors:** Yujian Jiang, Lin Song, Junming Zhang, Yang Song, Ming Yan

**Affiliations:** 1 State Key Laboratory of Media Convergence and Communication, Communication University of China, Beijing 100024, China; 2 Key Laboratory of Acoustic Visual Technology and Intelligent Control System, Ministry of Culture and Tourism, Communication University of China, Beijing 100024, China; 3 Beijing Key Laboratory of Modern Entertainment Technology, Communication University of China, Beijing 100024, China; 4 School of Information and Communication Engineering, Communication University of China, Beijing 100024, China

**Keywords:** sEMG, IMU, hand gesture recognition, convolutional neural network, recurrent neural network, transformer, residual networks

## Abstract

Gesture recognition based on wearable devices is one of the vital components of human–computer interaction systems. Compared with skeleton-based recognition in computer vision, gesture recognition using wearable sensors has attracted wide attention for its robustness and convenience. Recently, many studies have proposed deep learning methods based on surface electromyography (sEMG) signals for gesture classification; however, most of the existing datasets are built for surface EMG signals, and there is a lack of datasets for multi-category gestures. Due to model limitations and inadequate classification data, the recognition accuracy of these methods cannot satisfy multi-gesture interaction scenarios. In this paper, a multi-category dataset containing 20 gestures is recorded with the help of a wearable device that can acquire surface electromyographic and inertial (IMU) signals. Various two-stream deep learning models are established and improved further. The basic convolutional neural network (CNN), recurrent neural network (RNN), and Transformer models are experimented on with our dataset as the classifier. The CNN and the RNN models’ test accuracy is over 95%; however, the Transformer model has a lower test accuracy of 71.68%. After further improvements, the CNN model is introduced into the residual network and augmented to the CNN-Res model, achieving 98.24% accuracy; moreover, it has the shortest training and testing time. Then, after combining the RNN model and the CNN-Res model, the long short term memory (LSTM)-Res model and gate recurrent unit (GRU)-Res model achieve the highest classification accuracy of 99.67% and 99.49%, respectively. Finally, the fusion of the Transformer model and the CNN model enables the Transformer-CNN model to be constructed. Such improvement dramatically boosts the performance of the Transformer module, increasing the recognition accuracy from 71.86% to 98.96%.

## 1. Introduction

Human–computer interaction (HCI) is the study of information exchange and the mutual influence of technology between humans and computers. The Gesture Recognition System is one of the crucial components of the HCI system. Many wearable devices with human–computer interaction functions have been released in recent years. For example, Thalmic Lab’s Myo armband is a wearable device that can collect surface electromyography (sEMG) and Inertial Measurement Unit (IMU) and wirelessly transmits the two kinds of data to a server via Bluetooth signals. Since gesture recognition data are generated through sensors attached to the skin, the recognition results are not susceptible to light changes and object occlusions in the environment when using the Myo armband. Compared to gesture recognition in computer vision, the Myo-based gesture recognition method has become a hot topic of research in the field of HCI.

Many studies have proposed machine learning or deep learning algorithms to implement Myo-based gesture recognition tasks. Among them, support vector machine (SVM) [1,2,3,4], k-nearest neighbor (KNN) [5,6,7,8,9], decision tree (DT) [10], convolutional neural network (CNN) [11,12,13,14,15,16,17], recurrent neural network (RNN) [18,19,20,21,22,23,24], and artificial neural networks (ANN) [25,26,27,28,29,30] are the most popular algorithms with good recognition accuracy; however, there are still some challenges in this field of research. First, most studies build their datasets for specific application scenarios; these datasets involve mostly less than 10 gesture actions, and there is a lack of publicly available datasets for more classification tasks. Second, most existing studies only identify the sEMG signal during hand motion, and very little related research applies both sEMG and IMU to gesture action recognition. In addition, none of these studies evaluate the application of the Transformer model [31] to gesture recognition. Finally, for multi-gesture recognition tasks, there is still potential for research on building deep learning models with high accuracy and low time consumption for classification. The multi-gesture recognition should consider both dynamic and static arm and finger gestures. Hence, to meet the demands of the multi-gesture interaction scene, multiple deep learning models or their combinations are adopted to establish models for multi-gesture recognition with high accuracy and less time-consuming.

In this paper, the CNN-based, RNN-based, and Transformer-based two-stream gesture recognition models are proposed, respectively. Our self-built 20-category gesture dataset, including sEMG and IMU signals, far exceeds the normal 5-category and 6-category gesture datasets. According to the difference in characteristics between sEMG and IMU data for sampling frequency and data length, two-stream architecture is adopted to build basic CNN, RNN, and Transformer models. All of them aim to classify these 20 gestures at the same time, including both static and dynamic gestures. Afterward, the CNN-Res model, RNN-Res model, and Transformer-CNN model are established based on those three basic models. All of the improved models yielded exciting experimental results. By comparing the built models according to the experimental results, this paper selects the most suitable models to cope with different application scenarios for the best gesture recognition performance.

## 2. Related Work

The task of gesture recognition is to build a robust gesture recognition model with the acquired gestures data to obtain the ideal recognition results; it aims to contribute to the application in various human–computer interaction scenarios. After manufacturing a customizable wearable 3D-printed bionic arm that can be applied to amputees, Said S [1] successfully used the SVM to control the artificial bionic hand. In ref. [21], Nasri N took the Conv-GRU model as the classifier and created an sEMG-Controlled 3D game for rehabilitation therapies; moreover, the random forest (RF) model was also utilized by Mendes [32] to recognize Brazilian Sign Language in Sign Language recognition systems.

As mentioned in the introduction, most deep learning methods are based on the sEMG signals. The mainstream methods can be classified into three categories: CNN models [11,12,13,14,15,16,17], RNN models [18,19,20,21,22,23,24], and ANN models [25,26,27,28]. The single-layer CNN proposed by Zia ur Rehman M accomplished the classification task of 7 gestures [12], which pioneered the application of CNN models to gesture recognition. Ulysse Côté-Allard further optimized the basic CNN model and proposed a CNN model based on transfer learning in [13], resulting in the enhancement of the classifier’s performance in the recognition task of 7 gestures with higher accuracy. Similarly, the five-layer CNN model in [13] achieved good classification results based on sEMG signals.

Since the sEMG is a temporal signal, RNN classification models can also play a significant role in the classification based on sEMG. The RNN models applied to gesture recognition include the long short term memory (LSTM) and the gate recurrent unit (GRU). Nadia Nasri first introduced the GRU model to the 6-classification task of sEMG signals in [18], showing the feasibility of using recurrent neural networks to classify the data collected by the Myo armband. Zhen built a 21-classification sEMG dataset [20] and made it publicly available, which is a rare dataset for gesture recognition with more than 20 classifications in existing research. In [20], Zhen proposed a two-layer GRU model connected with fully connected layers. Although the average classification accuracy of the model is only 89.6%, Zhen strongly promotes the progress of GRU model application in multi-classification tasks. Additionally, some researchers have applied the recurrent-convolution neural network (RCNN) model proposed by Lai S for text classification [33] to classify sEMG signals. For example, Nadia Nasri [21] improved the former GRU model by adding a convolutional layer and improved the accuracy in a 7-classification task of sEMG signals, which implies that the combination of CNN models with RNN models can further extract the features of sEMG signals and strengthen the training efficiency of deep learning models. Currently, the Transformer model [31] has not been used in any research on gesture recognition.

With increasing categories in gesture recognition, the rising similarity between each gesture makes the classification more difficult. Capturing sEMG signals alone to recognize gestures seems not enough. Ulysse Côté-Allard, for instance, applied the CNN model to the task of a dataset with 11 classifications [16] but obtained test accuracies that were clearly lower than his previous results in 7 categories [13]; thus, naturally, some researchers are attracted to another signal captured by the Myo armband, the IMU signal. The IMU signal contains motion information and reflects the position variation characteristics during the execution. For illustration, Chiu [34] provided a thresholding method to determine the active signal segment of the motion. In his work, the IMU data were fed into a Long Short-Term Memory (LSTM) network for classification, demonstrating that there are possibilities existing in research for gesture recognition training through IMU signals.

Some scholars have begun to pay attention to combining sEMG and IMU signals for gesture recognition by machine learning algorithms [2,4,34,35] or deep learning algorithms [23,24]. Xiaoliang [24] imposed the LSTM model to solve the gesture recognition problem based on the combination of sEMG and IMU signals. Although Xiaoliang achieved to complete the classification task of 10 gestures, the accuracy remains to be uplifted. Williams [24] also proposed the RCNN model to settle the 5-class task with these two signals, reaching an accuracy of 99%. Both Xiaoliang and Williams took several wearable devices to obtain more adequate data, such as Smart Glove or several Myo armbands. The usage of more devices in the data acquisition was reasonable but inconvenient and limited the number of practical applications.

In conclusion, most studies on gesture recognition based on bioelectrical signals only built datasets of sEMG signals. When facing multiple gesture classification tasks, existing deep learning approaches with high gesture recognition accuracy are still not powerful enough to meet the practical needs of complex interaction situations. How to build a dataset with more variety of gestures and how to build a deep learning model that achieves multi-gesture classification with great precision is the target of future work in most related studies.

Inspired by the relevant work, a 20-class gesture dataset is constructed to meet the demands of multi-classification gesture recognition. For high accuracy and efficiency, the CNN-Res, RNN-Res, and Transformer-CNN models are proposed.

## 3. Methods

This Section describes how our gesture dataset and deep learning models are constructed. First of all, Section 3.1 describes the construction progress of the dataset. Then, Section 3.2 describes the establishment of the basic models. Finally, Section 3.3 describes how to optimize and combine the basic models to build the improved models.

### 3.1. Construction of Dataset

#### 3.1.1. Acquisition Tool

The data resource was a wearable acquisition tool, the Myo armband. The data collected were recorded by a workstation, and a large light-emitting diode (LED) screen was used to display the gesture instruction video.

The Myo armband has eight electrode sensors that can capture sEMG signals from the skin surface. There is also an inertial measurement unit with a three-axis gyroscope, three-axis accelerometer, and three-axis magnetometer. Data captured is sent wirelessly to the workstation via built-in Bluetooth. 

**Figure 1:**
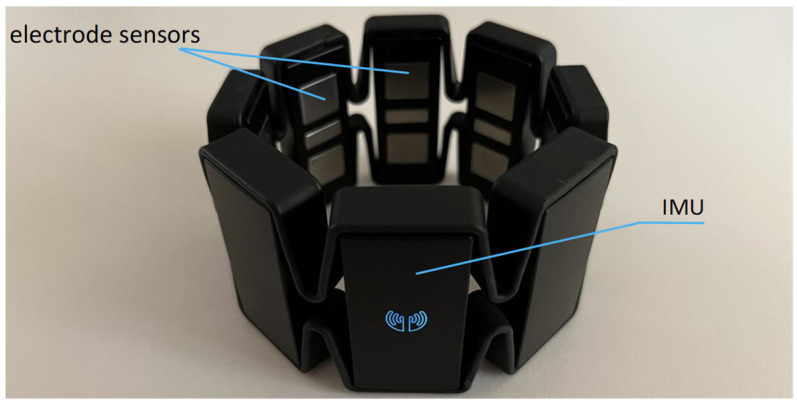
Outlook of Myo armband.

The position of sEMG’s electrode sensors and IMU is shown in Figure 1.

#### 3.1.2. Acquisition Process

**Figure 2:**
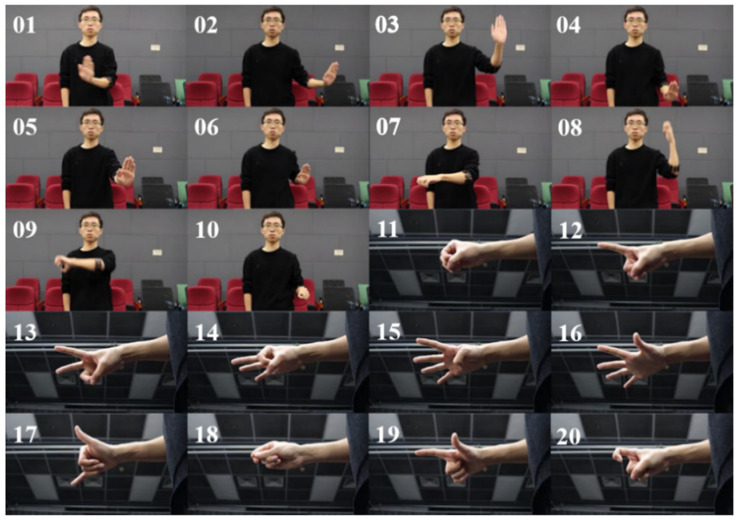
01~09 are dynamic movements. Their names are ‘Push left’, ‘Push right’, ‘Push up’, ‘Push down’, ‘Push forward’, ‘Push back’, ‘Turn left and right’, ‘Turn back and forth’, ‘Turn up and down’. 10~20 are static movements. Their names are ‘Making a fist’, ‘Number 0’, ‘Number 1’, ‘Number 2’, ‘Number 3’, ‘Number 4’, ‘Number 5’, ‘Number 6’, ‘Number 7’, ‘Number 8’, ‘Number 9’ in order.

We collected sEMG and IMU signals generated during gesture execution and designed 20 gestures, including translation in 6 directions, rotation in 3 directions, making a fist, and numbers 0~9. As shown in Figure 2, the former nine movements are dynamic, and the last 11 movements are static. Dynamic gestures contain an activation gesture. During the execution of dynamic gestures, the activation gesture is maintained all the time.

**Figure 3:**
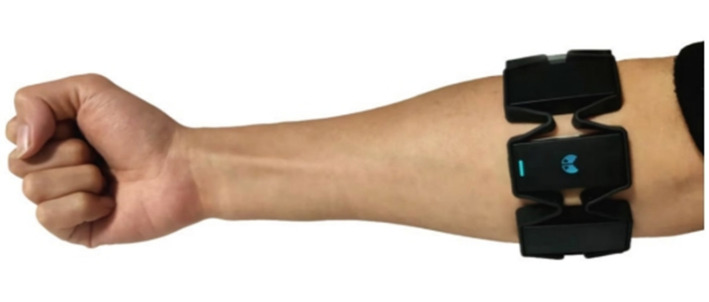
Position of Myo armband.

Fifty volunteers were recruited to participate in the experiment, all healthy undergraduate or graduate students. Among them, 14 were male, and 36 were female. All volunteers wore the Myo armband in the same position presented in Figure 3. The IMU was located on the medial side of the arm.

The volunteers were asked to make gestures following the recorded video of gesture instructions. Each gesture was performed once and took 5 s, and the interval between each gesture was 5 s. Following instructions on the video, the volunteers were prepared to carry out the gestures at the appropriate rhythm. The experiment started after ensuring that the volunteers were familiar with the gestures. To avoid muscle fatigue caused by the long-time performing the movements, the experiment was divided into two groups. The first group collected ten gestures and the second group collected another ten gestures, with a 5–10 min rest break in both groups. During the experiments, we checked the quality of completed gestures and the recorded data. The volunteers would be asked to re-capture the data after the experiment when there were unqualified movements and abnormal data. 

The actual time to complete the translational and rotational movements for dynamic gestures was within 2 s and 4 s, respectively. For static movements, all movements were kept within 5 s. Therefore, we only kept the valid length of each type of gesture to guarantee that all the gestures could be correctly classified. At last, we got 400 actions per volunteer, for a total of 20,000 actions. After eliminating the data of 271 incorrectly executed actions, the data of 19,729 gestures were stored in our dataset.

The sEMG data acquired is 8-channel with a sampling frequency of 200 Hz, while the IMU data obtained is 6-channel with a sampling frequency of 50 Hz. All data are sent by Bluetooth to the server and saved in the Myo Data Capture tool.

#### 3.1.3. Data Labeling

We take the Plotly Express extension library in Python to visualize the waveforms of the collected data and manually label the data. Before labeling, we need to get a clear waveform to distinguish a gesture’s start and end points to define the signal’s active segment.

First, we do full-wave rectification on the sEMG signal so that the data takes absolute values. That makes the negative half axis data roll over to the positive half axis. Then, we sum the data of all the channels (n = 8) and find the arithmetic square root of the sum as the new channel, as shown in function (1); these two operations are intended to display the 8-channel sEMG signal in one waveform plot and reduce the dispersion of the amplitude of it; this new waveform is then smoothed with a moving average algorithm. Each data point is replaced by the average of M/2 points before and after (including the point itself), as shown in function (2). Smoothing is applied to obtain a more intuitive waveform diagram, which is convenient for our subsequent labeling.

(1) $$x_{sum}=\sqrt{\sum \left|x^{\left(k\right)}\right|},\;k=1,2,3,\;\ldots ,\;n$$

(2) $$x_{new}\left(i\right)=\frac{\sum_{i-\frac{M}{2}}^{i+\frac{M}{2}}x_{sum}\left(i\right)\;\;}{M+1},\;i=1,2,3,\;\ldots ,\;m$$

where n is the number of channels, m is the number of data points in the merged channel, $x^{\left(k\right)}$ is the data of each original channel,$\;x_{sum}$ is the data after merging channels. $x_{sum}\left(i\right)$ is each data point of the merged channel, M+1 is the number of data points used in each smoothing process, and $x_{new}\left(i\right)$ is the value after smoothing.

**Figure 4:**
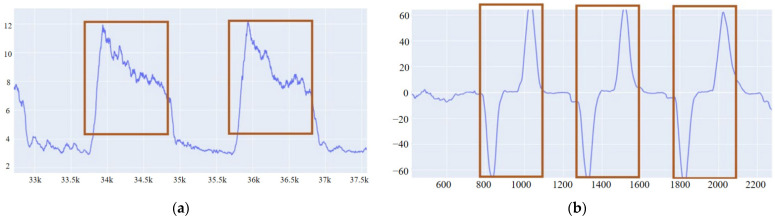
Active segments of sEMG and IMU. (**a**) sEMG, (**b**) IMU.

After our verification, the waveform is observed when M is taken as 50. As shown in Figure 4a, the blue line represents the value of the signal, and the orange box is the active segment of a signal which is also the data segment we need to label. The corresponding amount of data is taken to label the segments according to the sEMG data length for the different gestures. Labels of the gesture category are 1–20.

For the signals measured by IMU, their six channels are processed by the moving average algorithm in function (2), respectively. Furthermore, it turns out that the data of the y-channel of the gyroscope is relatively easy to identify, as shown in Figure 4b. Therefore, there is no need to merge channels, and the data of the y-channel can be directly regarded as the labeling basis of the signal activity segment. Depending on the length of IMU data for different gestures, the corresponding amount of data is taken. Labels of the gesture category are 1–20, the same as for the sEMG data.

#### 3.1.4. Data Segmentation

The gesture movements are continuous. To meet the real-time requirements of gesture interaction, the active segment data should be segmented with a sliding window.

According to Mendes [32], the sliding window length is selected as 1 s (containing 200 points for sEMG and 50 points for IMU) with a sliding step of 100 ms to segment the sEMG and IMU signals. Eventually, 601,489 sEMG samples of size 200 × 8 and 601,489 IMU samples of size 50 × 6 are generated.

Before inputting data into the model, we conducted the normalization operation. Since the sEMG signal and IMU signal differ in values and the IMU signals contain two types of data: accelerometer and gyroscope, we normalized the sEMG and IMU between $\left(-1,1\right)$ with function (3), respectively.

(3) $$x^{\prime }\left(j\right)=2\times \frac{x\left(j\right)-x_{min}}{x_{max}-x_{min}}-1,j=1,2,3,\ldots N\;$$

where N refers to the number of data points in the sEMG or IMU signal. $x_{min}$ refers to the minimum value of sEMG or IMU signal. $x_{max}$ refers to the maximum value of the sEMG or IMU signal. $x\left(j\right)$ refers to the value of each data point. $x^{\prime }\left(j\right)$ refers to the value of each data point after normalization.

### 3.2. Basic Models

CNN and RNN are the most common models in gesture recognition research; however, the Transformer has not been introduced in any associated research so far. In this Section, a two-stream CNN model, a two-stream RNN model, and a two-stream Transformer model are built to classify the dataset to test the feasibility of these basic models for the sEMG and IMU-based gesture recognition missions.

#### 3.2.1. Two-Stream CNN Model

The basic CNN consists of a convolutional layer, a pooling layer, and a fully connected layer.

To transform the data into a form suitable for a 2D convolutional kernel, dimensional change should be performed on each data. First, the channel dimension is retained, and only the first 196 data points of the sEMG and the first 49 data points of the IMU are reserved. Then, we reshape these points into data of size 14 × 14 and 7 × 7, respectively. After that, the size of sEMG data is changed to 14 × 14 × 8, and the size of IMU data is transformed to 7 × 7 × 6.

**Figure 5:**
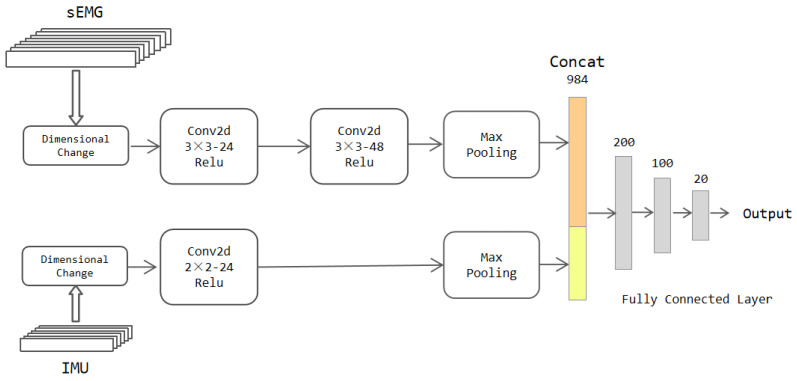
The architecture of the two-stream CNN model.

As shown in Figure 5, the sEMG and IMU data are divided into two streams. In turn, there is a convolutional layer, a maximum pooling layer, and a convolutional layer in the first stream. A Relu layer follows each convolutional layer. The first and second convolutional layer contains 24 and 48 filters of size 3 × 3, respectively. The maximum pooling layer has a window size of 2 × 2, which can compress the data size while preserving the key features. The second stream has a convolutional layer and a maximum pooling layer. The convolutional layer also consists of 24 filters of size 3 × 3, connected to a Relu layer. The pooling layer also has a window of size 2 × 2. The outputs of two streams are expanded into a single column and are concatenated together after passing through the maximum pooling layer. The output of the last fully connected layer is the classification result corresponding to the input data.

#### 3.2.2. Two-Stream RNN Model

The LSTM [36] and the GRU [37] models are the most frequent RNN models. For the LSTM model, there are three ways of transferring information between neural units: the long-term information of the previous moment, the output information of the previous moment, and the input information of the current moment; these three information paths are controlled by the forgetting gate, the input gate, and the output gate. The GRU model [37] is a variation of the LSTM model. GRU optimizes the three gates of the LSTM into an update gate and a reset gate, and these two gates control the hidden state of the current moment and the post-selected hidden state, respectively. The GRU model has fewer parameters and a faster training time than the LSTM model; moreover, in many kinds of application cases, GRU can achieve comparable results to LSTM.

**Figure 6:**
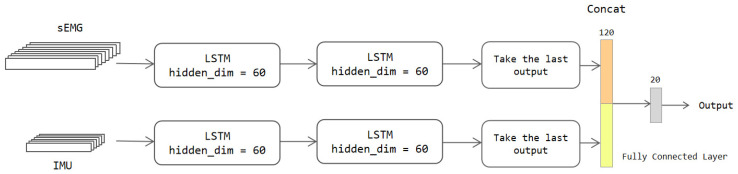
The architecture of the two-stream LSTM model.

We take the two-stream LSTM model as an example to introduce the RNN architecture. As shown in Figure 6, the data are directly input to the two-stream LSTM model in two streams. Each stream has two layers of LSTM network with 50 hidden units. The hidden layer of the LSTM network can capture the state information of the data in the time dimension. We take the information output from the last time node of the LSTM as a vital feature for the classification. The features output from the two-stream network are also expanded into a column and then concatenated together. The fully connected layer has 20 neurons and can be utilized to output classification results depending on the training dataset. 

The two-stream GRU model is formed by replacing the LSTM layer in the two-stream LSTM model with the GRU layer. The structure and parameter settings of the model are the same as those of the two-stream LSTM model.

#### 3.2.3. Two-Stream Transformer Model

Transformer is a very innovative network proposed by Google Brain in [31], which abandons the circular structure and employs a more interpretable self-attention mechanism to extract the relationships between data. The self-attention mechanism involves three quantities: Query, Key, and Value; they aim to compute the relationship between input data X. 

Query represents the query vector, Key represents the vector of the relevance of the queried information to other information, and Value represents the queried information vector. The specific calculation is shown in function (4).

(4) $$\left\{\begin{array}{c} Q=XW^{Q}\\ K=XW^{K}\\ V=XW^{V} \end{array}\right.$$

where Q refers to Query. K refers to Key. V refers to Value. X refers to the input data. $W^{Q}$, $W^{K}$, and $W^{V}$ are the weight matrices, updated with the training, corresponding to these three quantities.

After the values of the three vectors Query, Key and Value are calculated, the self-attention mechanism computes the eigenvalues of the output of the attention layer with function (5).

(5) $$Attention\;\left(Q,\;K,\;V\right)=softmax\left(\frac{QK^{T}}{\sqrt{d_{k}}}\right)V$$

where $d_{k}$ refers to the dimension of the vector K.

For more connections among the input data, the Transformer extracts features of the input data through a multi-headed attention mechanism. The multi-head attention mechanism consists of multiple self-attention layers, and each self-attention layer is computed in parallel. Then, the output of each head is spliced and multiplied by the weight matrix. The specific calculation is shown in function (6) and function (7).

(6) $$MultiHead\left(Q,\;K,\;V\right)=Concat\left(head_{1}\right.,\ldots ,\;head_{h})W^{O}\;$$

(7) $$where\;\;head_{i}=Attention\left(QW_{i}^{Q},KW_{i}^{K},\;VW_{i}^{V}\right)\;$$

**Figure 7:**
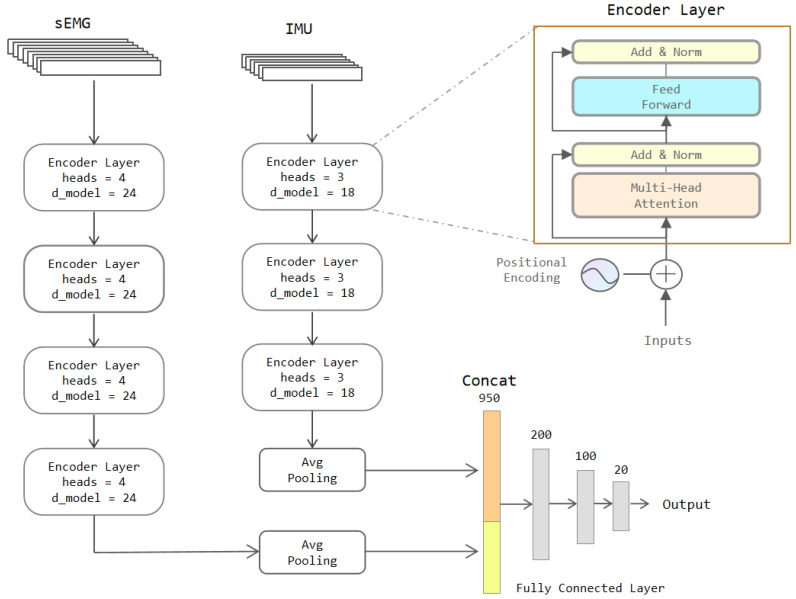
The architecture of the two-stream Transformer model.

The Encoder module in the Transformer model is applied in our model to extract the features of the sEMG and IMU signals. The input is first positional encoded so that it has the sequential position information; it subsequently enters a multi-headed attention layer to compute the correlation features and an Add&Norm layer consisting of a residual layer such as ResNet [38] and a normalization layer. Then it proceeds to the Feed Forward layer with two linear units. The Feed Forward layer strengthens the expressiveness of the model, and the output has the exact dimensions as the input. The next is the same Add&Norm layer as the previous one. In the Encoder Layer, the number of heads in the multi-attention mechanism and the number of hidden neural units (d_model) of the FF layer are parameters that can be defined. The complete model is shown in Figure 7.

As presented in Figure 7, the sEMG data pass through 4 layers of Encoder Layers to retrieve features, followed by the average pooling layer. The average pooling layer is a 1D pooling layer with a window length of 2. That means the features are compressed to half of their original size. The IMU data also pass through 3 Encoder Layers. The features are also squeezed to half the initial size by the same pooling layer. The outputs of these two pooling layers are stitched together and fed to the three fully connected layers to output the classification results.

### 3.3. Improved Models

At first, the two-stream CNN is regarded as the base model, and a residual network is applied to build the CNN-Res model. Next, the two-stream RNN model is combined with the CNN-Res model to construct the two-stream LSTM-Res model and the two-stream GRU-Res model. Finally, the two-stream Transformer model is fused with the CNN model, forming the two-stream Transformer-CNN model.

#### 3.3.1. Two-Stream CNN-Res Model

In the basic CNN model, only one or two convolutional layers are extracting features of sEMG and IMU data; it is prone to insufficient extraction of features when the number of convolutional layers is small. More layers are a necessity for retrieving more features of significance.

**Figure 8:**
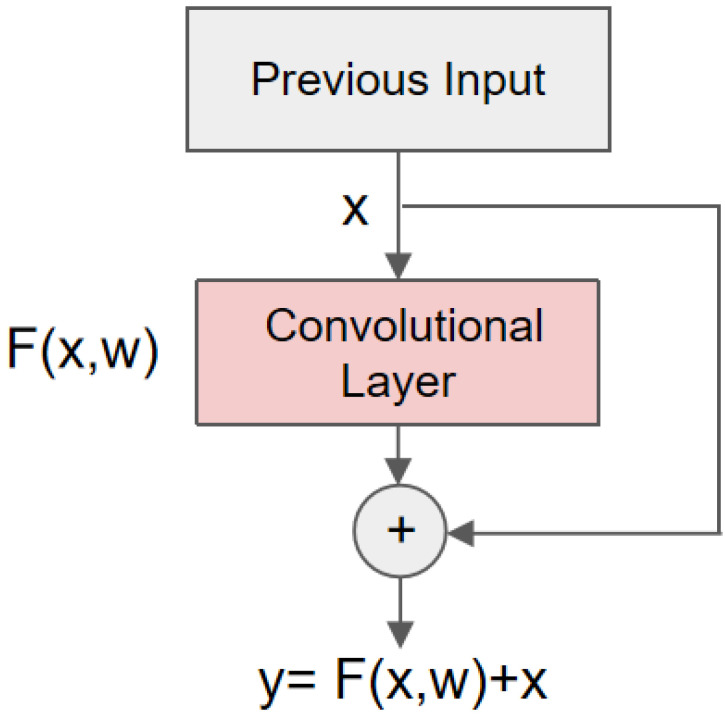
The structure of a Residual unit.

Providing the network is deeper, there is an exposure of gradient disappearance or gradient explosion. At the same time, the deeper network may also trigger the problem of network degradation. Therefore, the residual network [38] is introduced into our network to prevent degradation. A standard residual unit is shown in Figure 8.

The layer-hopping connection in the residual network allows the input signal to propagate directly from any lower layer to a higher layer; it enables the network to converge faster during training. Deepening the network layers often comes at the cost of increased training time. Thus, the introduction of residual networks compensates for the drawback of a longer training time for deeper networks to some extent.

**Figure 9:**
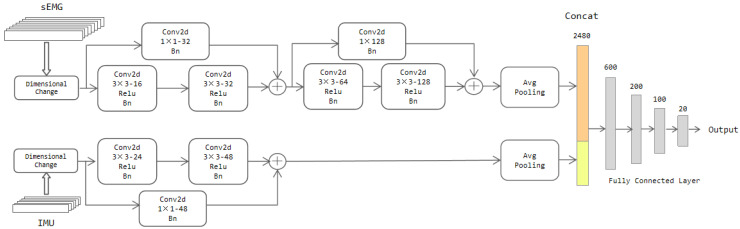
The architecture of the two-stream CNN-Res model.

Motivated by the above analysis, a two-stream CNN-Res model is proposed, and its specific architecture is shown in Figure 9. The data are also input to the network after the dimensional change mentioned in Section 3.2. The first stream network is fed with the sEMG signal and the second stream with the IMU signal.

The parameter settings in the architecture were obtained through tweaking, where we wanted to find a model with high recognition accuracy and relatively low complexity. For the number of network layers, we chose a range from two to six and found that when it was four for the first stream and two for the second stream, the complexity and accuracy of the model reached a balanced state. Two strategies were tried out for the number of channels, namely keeping it constant in all layers or doubling it when going from one layer to the next deeper one; it turned out that the recognition accuracy of the latter was better than the former; thus, after having fixed this strategy, the only choice we had to make was determining the number of filters in the first convolutional layer. Considering the differences in input channel numbers of the two streams, they had better be selected in different ranges. Therefore, we chose them from 4 to 24 and 8 to 48, respectively. Within these ranges, taking 16 for the first and 24 for the second was the optimal choice. For convolution kernel size, 2 × 2 and 3 × 3 were tried, and 3 × 3 was chosen.

The mainstay of the first stream network is composed of 4 convolutional layers and an adaptive averaging pooling layer. Each convolutional layer contains a different number of filters of 3 × 3, but all of them have a Relu layer and a batch normalization (bn) layer. The filters of these four convolutional layers are 16D, 32D, 64D, and 128D, respectively. The second stream backbone encompasses two convolutional layers with the same content as the first stream network. The number of filters in these two convolutional layers is 24 and 48, respectively.

After every two convolutional layers, a residual unit is added. To ensure the number of channels between the data at the residual cells matches well, we equip a convolutional layer with a 1 × 1 convolution kernel to change the number of channels of the previous input. Padding operation is also applied in every layer of convolution. The objective is to prevent the data size from changing with the convolution operation so that the data size has consistency. At the output of the two feature streams, they are squeezed by the adaptive average pooling layer to size 128 × 4 × 4 and 48 × 3 × 3, respectively. The maximum pooling layer is not applied here. The rationality is that the average pooling layer can retain more information compared to the maximum pooling layer. The squeezed features are stretched into a column and then stitched together into four fully connected layers to get the classification results.

#### 3.3.2. Two-Stream RNN-Res Model

We built the two-stream RNN-Res model by combining the two-stream RNN model with the two-stream CNN-Res model.

For sEMG and IMU signal data, the LSTM and the GRU are good at extracting long-term dependence features that reflect the global significance of each data point. On the other hand, CNN is a network adept at extracting local features of the data [12]; thus, the CNN network is placed behind the RNN, allowing for further extracting the local features of these long-term dependence features. The combination of long-term dependence features and local features improves the model’s characterization ability. Features output by the LSTM and GRU models have already reflected the data’s features in the temporal dimension; thus, the 2D convolutional layer in the CNN model does not lose the location information in the original data.

**Figure 10:**
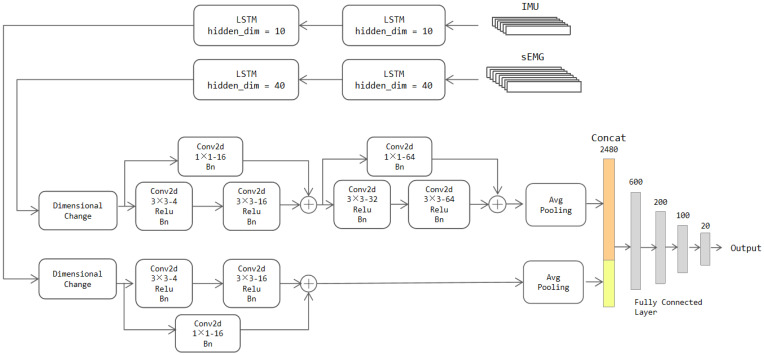
The architecture of the two-stream LSTM-Res model.

The 2D convolutional layer cannot directly operate on the RNN model output form. Therefore, there is a dimensional transformation operation in front of the CNN model. A dimension of size one is inserted before the temporal dimension of the output features; this action not only preserves original features’ content and order but also makes it extracted in the correct form by the CNN-Res network. Furthermore, to increase the depth of the model while reducing the time required for convergence during training, we equally add a residual network to the CNN. The specific network structure is shown in Figure 10.

The LSTM module has the same structure as Section 3.3.1; however, considering that the LSTM-Res model is complicated, we make the following improvements to reduce the complexity of the network:

(a) Hidden units in the first stream reduce from 60 to 40 and in the second stream from 60 to 10.

(b) Inter-layer maximum pooling layers to the CNN model for down-sampling are added. Their window sizes of the first stream are set to 10 × 2 and 2 × 2 after the second and fourth convolutional layers, respectively. Similarly, a maximum pooling layer of size 5 × 1 is placed after the second convolutional layer in the second stream; these pooling layers squeeze the size of data transmitted.

The rest of the structure in the CNN block is almost consistent with the CNN-Res model. The parameter tweaking was also conducted according to the parameter tweaking process of the CNN-Res model; however, the data’s channel numbers of the two streams are both one after the dimensional change, so the number of initial convolution kernels at their starts are chosen to be the same. After tweaking, we choose the combination of “4D, 16D, 32D, 64D” for filters in the first stream and “4D, 16D” in the second stream. After the same adaptive pooling layer, the two streams are concatenated together, and then the classification results are output by the fully connected layers.

As for the GRU-Res model, its architecture is the same as the LSTM-Res model, except for the different RNN network types.

#### 3.3.3. Two-Stream Transformer-CNN Model

In the Transformer, matrix computation eliminates the need for step-by-step computation to obtain features for long-distance data; however, this feature extraction method ignores local details, making the Transformer less capable of capturing local features. In contrast to text information, the sEMG and IMU signals are continuous data generated during action execution. Therefore, ignoring local details can lead to inadequate extraction of features in the temporal dimension of the data.

To enhance the model’s ability to extract local features, we improve the Transformer model as follows:

(a) A 1D convolutional layer is added between each encoder layer; these convolutional layers not only extract local features but also increase the number of channels of features. Each convolutional layer doubles the number of channels.

(b) The values of heads and $d_{\_}model$ in each encoder layer are changed with the deepening of the network. The increase of parameters allows the model to extract deeper global information as the number of network layers deepens.

**Figure 11:**
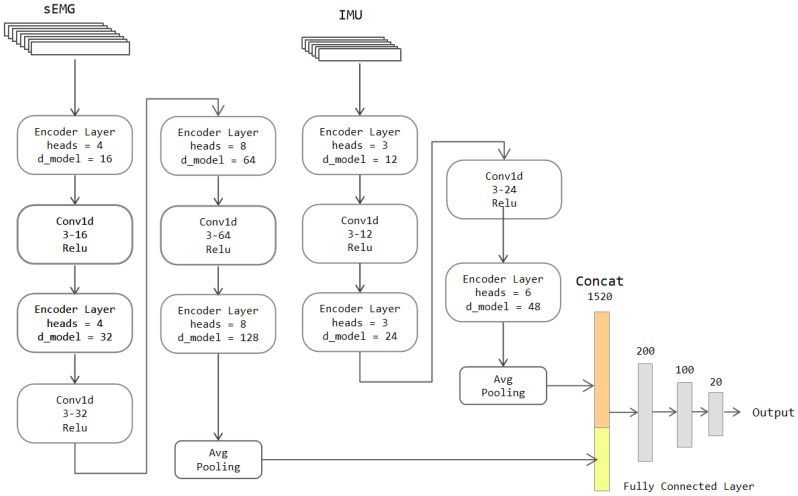
The architecture of the two-stream Transformer-CNN model.

The above modifications strengthen the model’s ability to extract local features as well as increase the information contained in the global features. Given that the convolutional layer’s outbound features must be operated by Encoder Layers, temporal messages of the data cannot be destroyed. That is why we take the 1D convolution to obtain local features instead of the 2D convolution. The convolutional layer also contains a padding operation to keep the data length from changing with convolution. To avoid corrupting the location information of the features, we also use an average pooling layer instead of a maximum pooling layer. The amount of information on the features is kept to the maximum extent. The sEMG and IMU data are also input as two streams, and the specific network structure is shown in Figure 11. 

The parameter tweaking of the Transformer-CNN model mainly included: the value of heads and ${\;d}_{\_}\mathrm{model}$ in Encoder Layers and the numbers of filters in 1D convolutional layers. The value of heads must be divisible by the input channel numbers and should not be too large. The choices contained 2, 4, and 8 for the first stream and 3 and 6 for the second stream. After our experiments, when the values of the heads were set to “4, 4, 8, 8” and “3, 3, 6”, the accuracy was the highest, and the complexity of the model was relatively low. The parameter settings of 1D convolutional layers were similar to the model defined in Section 3.3.1. We also decided to double the number of channels when signals passed through convolutional layers. The number of filters for the first convolutional layer was selected from 4 to 24 for the first stream and 8 to 48 for the second stream. According to our test results, the best choice is to take 16 for the first and 12 for the second; the tweaking of ${\;d}_{\_}\mathrm{model}$ was identical to them.

The average pooling layer of the first stream has a sliding window length of 10, and the average pooling layer of the second stream has a sliding window length of 5. After the pooling operation, the length of the sEMG data is compressed to 20. The length of the IMU data is shortened to 10. The features of these data are concatenated and input to a four-layer fully connected network to get the classification outcome.

## 4. Experiments and Results

This Section introduces our experiments and the results of our models. Not only was the experiment of simultaneous classification of 20-category gestures carried out, but also the sEMG signal and IMU signal were applied to identify arm movements and finger movements, respectively. To begin, Section 4.1 states our experimental conditions. Then, Section 4.2 states 20-category experimental results of our basic models, and Section 4.3 states 20-category experimental results of our improved models. After that, Section 4.4 states comparisons and analyses of results of basic models and improved models. Furthermore, Section 4.5 states the results of 9 types of arm movements and 11 types of finger movements, which were recognized separately by two signals.

### 4.1. Experimental Conditions

Classification experiments on our dataset are performed with the basic and improved models defined in Section 3. As samples generated from the dataset after data segmentation exceeded 600,000, the dataset was divided into training and test sets in the ratio of 49:1. 589,459 samples are in the training set, and 12,030 samples are in the test set.

All the models were trained with the training set and tested on the test set after training. The training loss function was the cross-entropy loss function, and the stochastic gradient descent method was the approach to update the model weights in all the experiments. The batch_size was set to 32. The learning rate was set to 0.005 for the two-stream RNN model and 0.001 for other models (including improved models). Additionally, all of the training processes for the experiments were performed by a GeForce RTX 3090 GPU for 100 epochs; it should be noted that the test set was not involved in updating the model parameters and was only available for testing the model recognition accuracy.

### 4.2. Results of Basic Models

The experiments of the base models were carried out first, and the results are shown in Table 1. Table 1 contains the training time, testing time, and testing accuracy of all the base models in the experiments of this paper. As can be seen from the table, the two-stream LSTM model and the two-stream GRU model have the highest test accuracy of 97.10% and 95.91%, respectively. The test accuracy of the two-stream CNN model reaches 95.43%. The test accuracy of the two-stream Transformer is the lowest, at 71.68%. 

It is evident that the two-stream CNN and RNN models complete the 20-classification task on our dataset with high accuracy, but there is still room for further improvement. Nevertheless, the basic Transformer model is far from satisfying our requirements for recognition accuracy.

### 4.3. Results of Improved Models

The experiments with improved models were then conducted, and the new results were included in Table 2; it shows an overview of all models’ training time, testing time, and accuracy. The ES refers to the experiment incorporating the Early Stopping mechanism into the training process. Single test time means how long it requires to classify one single sample.

As presented in Table 2, it is found that the test accuracy of the CNN-Res model achieves 98.24%; the test accuracy of the LSTM-Res and GRU-Res models reach 99.67% and 99.49%, respectively. Surprisingly, the test accuracy of the Transformer-CNN model attains 98.69%. That is considered sufficiently accurate for gesture recognition. Compared with the basic models, the improved models are significantly more excellent than the basic models. The recognition precision has been enhanced to different degrees. Among the results, the Transformer model has the most striking accuracy gain, about 27.28%.

Because of the improved models’ grown complexity, the training and test times naturally become longer; however, the introduction of residual units allows the convergence speed of model training to be significantly boosted, which means the Early Stopping can be implemented to shorten the training time. Because of the Early Stopping, the actual training time of CNN-Res, LSTM-Res, and GRU-Res is even shorter than the training time of the basic models, which is 1.43 h, 8.6 h, 11.7 h, respectively; it turns out that the CNN-Res model has the smallest training and test time among improved models. Therefore, it has the highest training and testing efficiency among all the models. As for the single test time, the Transformer-CNN has the shortest single test time, which means it holds the quickest recognition response.

### 4.4. Comparison and Analysis

The experimental performance of the improved and basic models will be compared by the variation of the models’ loss and test accuracy during the training process. Their test confusion matrixes are also drawn to analyze the details of each gesture recognition result. 

#### 4.4.1. CNN vs. CNN-Res 

**Figure 12:**
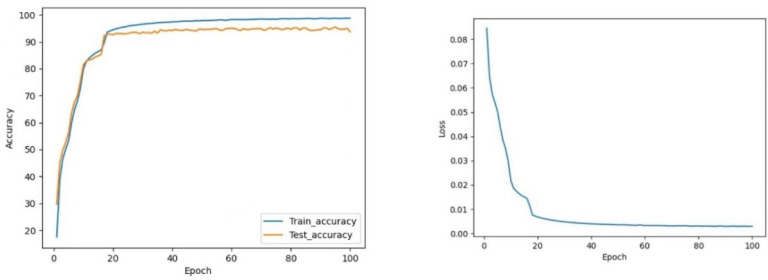
The curve of accuracy and loss during training of CNN.

**Figure 13:**
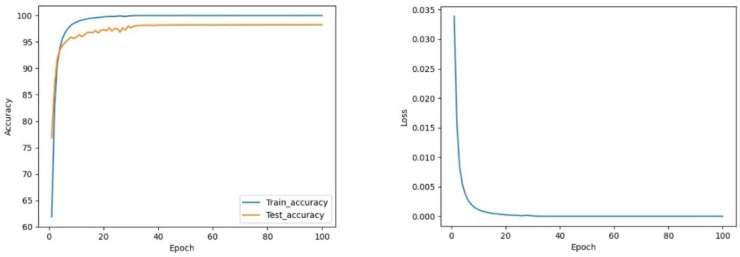
The curve of accuracy and loss during training of CNN-Res.

As shown in Figure 12 and Figure 13, the CNN model maintains the test accuracy of around 94% after stabilization, and the loss value stays within 0.004. In contrast, the accuracy of the CNN-Res model is significantly raised and can be maintained above 98.13% after stabilization; moreover, the Loss value also decreases significantly after stabilization and always stays within 0.001. Finally, at the 97th epoch, its test accuracy reaches the highest value of 98.25%.

The test accuracy graph indicates that the CNN-Res model’s training process converges at the 33rd epoch, which takes only about 1/3 of the entire training time. Thus, it can be seen that one primary advantage of the introduction of the residual network is that the CNN-Res model significantly improves the convergence speed of training when the network becomes deeper; its training efficiency is strengthened a lot.

**Figure 14:**
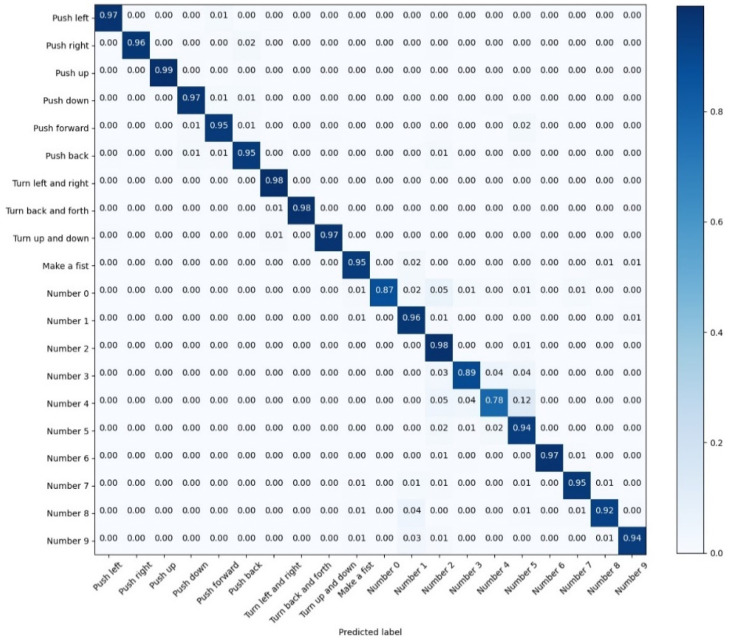
The confusion matrix for the test results of CNN.

**Figure 15:**
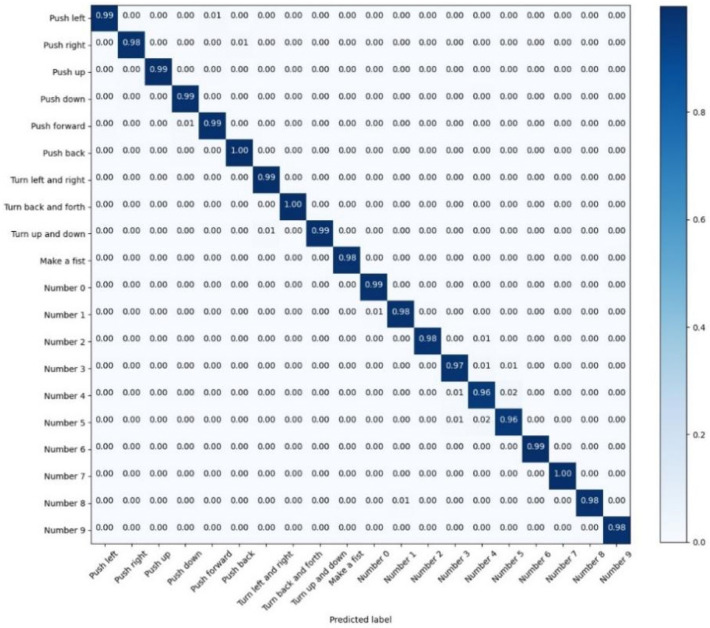
The confusion matrix for the test results of CNN-Res.

According to Figure 14, the recognition accuracy of various gestures is not uniform to the CNN model. The identification accuracy of ‘Number 0′, ‘Number 2′, and ‘Number 4′ is 87%, 89%, and 78%, respectively; this deficiency could be attributed to the similarity of these three static actions, which brings about more difficulties in recognition. As for the recognition results of the CNN-Res model as shown in Figure 15, the recognition accuracy of ‘Number 0′, ‘Number 2′, and ‘Number 4′ is enhanced to 99%, 98%, and 96%, respectively. Furthermore, the recognition accuracies of the other 17 actions are also promoted.

After expanding the layers and introducing residuals, the CNN-Res model achieves better feature extraction with a higher convergence speed.

#### 4.4.2. RNN vs. RNN-Res

**Figure 16:**
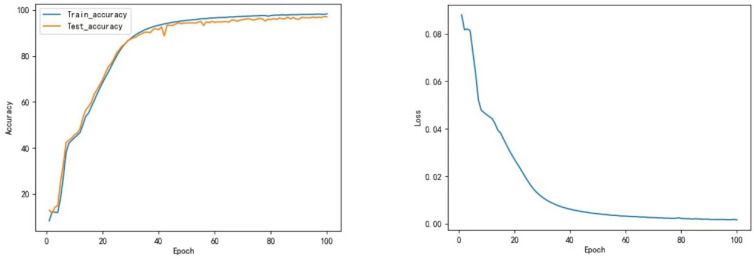
The curve of accuracy and loss during training of LSTM.

**Figure 17:**
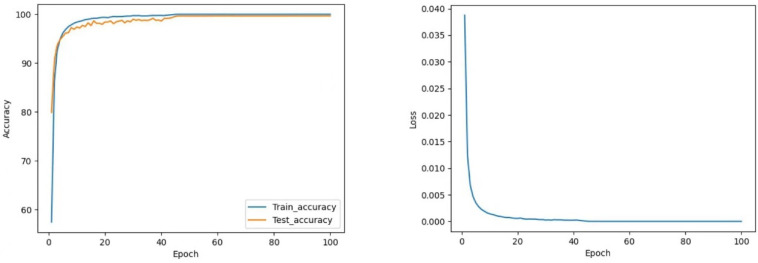
The curve of accuracy and loss during training of LSTM-Res.

We take the comparison of the LSTM model and LSTM-Res model as an example. As illustrated in Figure 16, the LSTM model keeps the test accuracy above 96% after stabilization, with the highest classification accuracy of 97.10%. Also, the Loss value is held within 0.003. The result suggests that the LSTM model already possesses a good classification effect. To our excitement in Figure 17, however, the test accuracy of the LSTM-Res model reaches up to 99.67% after being stabilized; moreover, the Loss value is almost 0 at a steady state.

What is more, the training of the LSTM-Res model is fully converged at the 46th epoch, accounting for only about 1/2 of the whole training time; it shows that after combining the CNN-Res model, the LSTM-Res model not only benefits the precision but also doubles the training convergence speed based on the LSTM model.

Due to the high test accuracy of LSTM-Res, we keep the values of the test confusion matrix of LSTM-Res with three decimal places.

**Figure 18:**
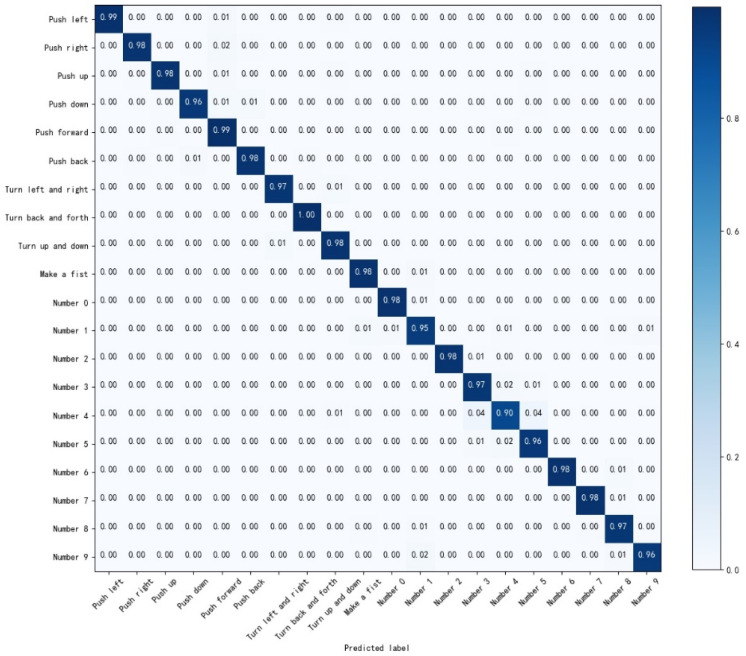
The confusion matrix for the test results of LSTM.

**Figure 19:**
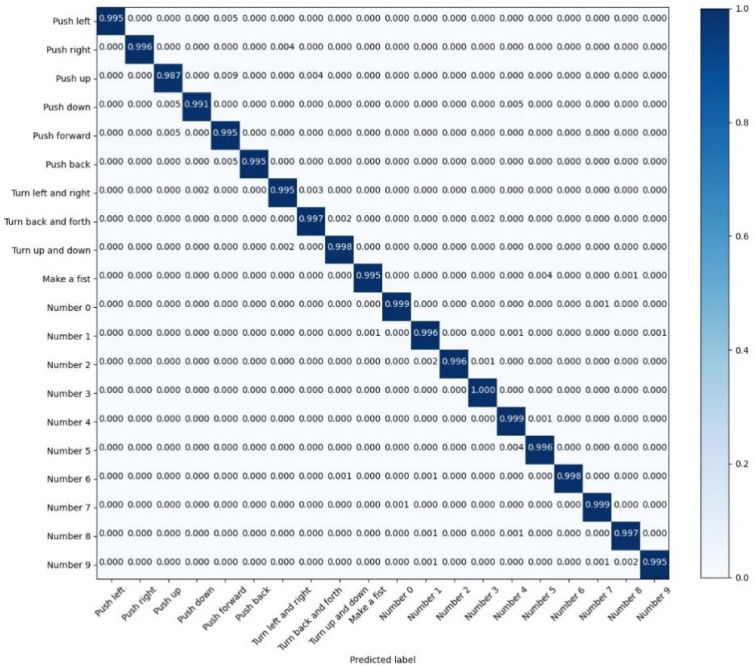
The confusion matrix for the test results of LSTM-Res.

Among the recognition results of the LSTM model in Figure 18, there are 19 gestures whose recognition accuracy has attained more than 95%; however, the recognition accuracy of the ‘Number 4’ is relatively low, which is 90%; it reveals that the LSTM model has achieved high accuracy in gesture recognition. Still, the accuracy of individual gestures is not high enough. Nevertheless, as exhibited in Figure 19, apart from “Push up”, the other 19 actions are classified with an accuracy of over 99%.

The performance improvement of the LSTM-Res model is credited to the CNN-Res model. The LSTM-Res model can accomplish the 20-classification task with outstanding accuracy and less training time by further extracting the local features with the CNN.

#### 4.4.3. Transformer vs. Transformer-CNN

**Figure 20:**
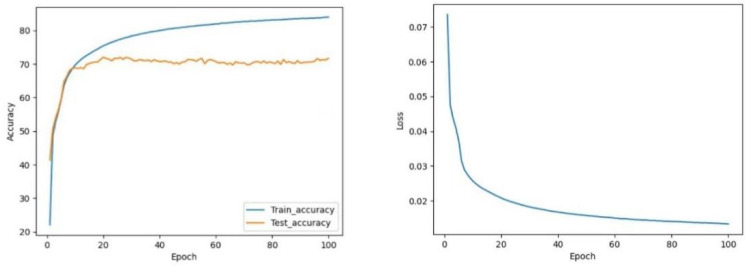
The curve of accuracy and loss during training of Transformer.

**Figure 21:**
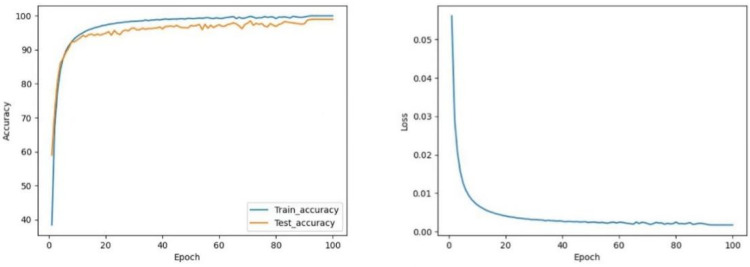
The curve of accuracy and loss during training of Transformer-CNN.

Figure 20 manifests that the Transformer model does not perform well in classification. Even after undergoing 100 epochs of training, the test accuracy is still only 71.68%; it indicates that the basic Transformer model for gesture recognition is far from sufficient in gesture recognition; however, with the CNN model’s fusion, the Transformer-CNN model’s accuracy is extensively promoted. As shown in Figure 21, the test accuracy of the Transformer-CNN model stabilized as high as 98.96%, but the convergence time did not change obviously.

**Figure 22:**
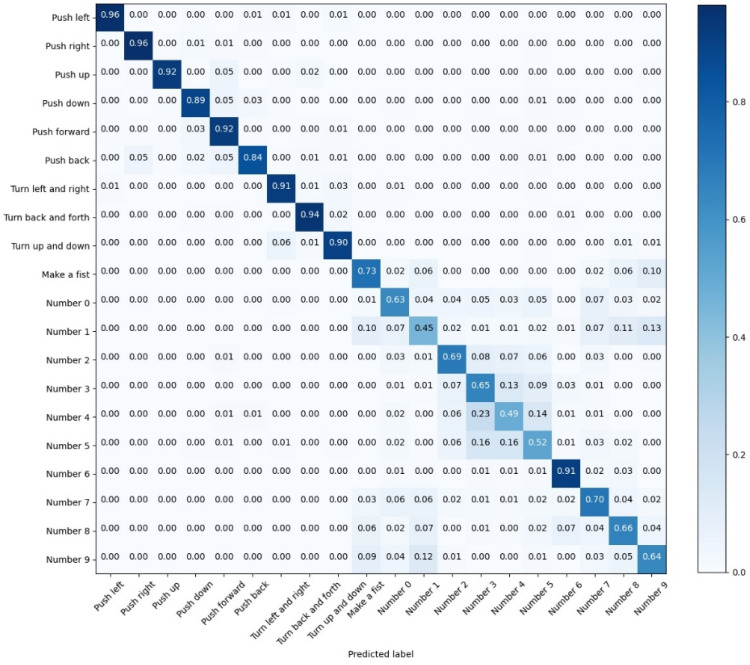
The confusion matrix for the test results of Transformer.

**Figure 23:**
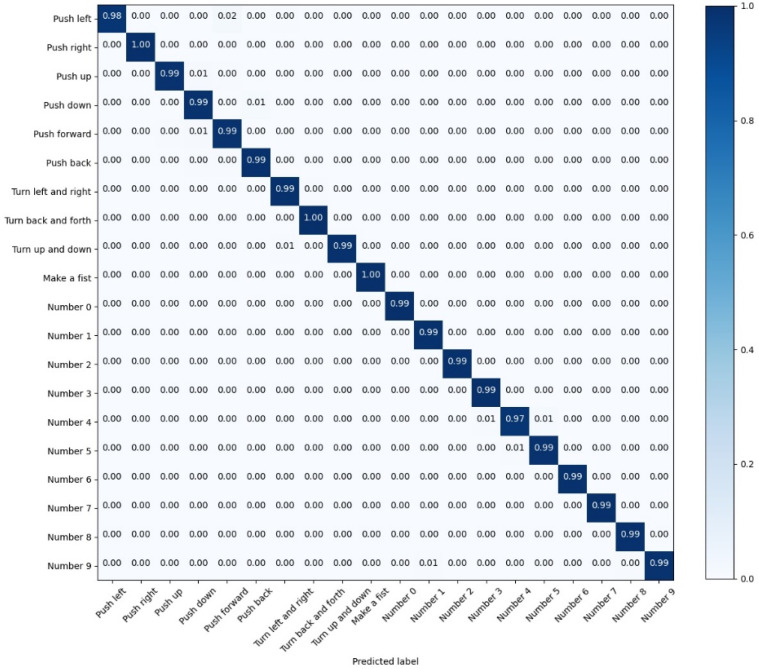
The confusion matrix for the test results of Transformer-CNN.

As shown in Figure 22, almost half of the gesture recognition accuracies are under 75%., not to mention that the recognition accuracy of ‘Number 1′, ‘Number 4′ and ‘Number 5′ is only about 50%, which means half of them are incorrectly recognized; however, the Transformer-CNN model’s confusion matrix is much higher quality. Figure 23 shows that the classification accuracy of almost all gestures is around 99%. Remarkably, three gestures are correctly identified with 100% accuracy. The lowest accuracy comes from ‘Number 4′, which is still 97%.

The above comparisons substantiate that the incorporation of the 1D convolutional module ameliorates the performance of the Transformer. As a result, it is apparent that the Transformer-CNN model behaves better than the Transformer model in the task of 20-gesture classification.

In summary, the LSTM-Res/GRU-Res model is preferable if the system’s objective is to achieve the highest recognition accuracy because they have the best precision; however, the CNN-Res model is the best choice if the system requires a high training efficiency and less testing time, not with a highly demanding requirement for accuracy; it has the least time for training and testing, and its recognition accuracy is up to 98.24%. On top of that, if the system needs high accuracy and the most rapid real-time response to a single gesture, the Transformer-CNN model is the ideal option because of the highest recognition speed and test accuracy of 98.96%. 

### 4.5. Separate Recognition

Our self-built dataset of 20 categories contains nine arm movements and eleven gesture movements; they can be divided into a 9-category sub-dataset and an 11-category sub-dataset.

To further explore the effect of sEMG and IMU signals on the recognition of arm and finger movements, we also split sEMG and IMU signals to identify arm and finger movements, respectively. The improved CNN-Res model, RNN-Res model, and Transformer-CNN model in Section 4.4 have been implemented in three groups of experiments. Each group includes using the sEMG signal to recognize arm and finger movements and the IMU signal to recognize arm and finger movements. The separated experimental results are shown in Table 3, where ‘Together’ represents the result of simultaneous recognition in Section 4.4.

Table 3 reveals that the three models can recognize arm movements with more than 90% accuracy when using sEMG or IMU signals independently; however, for finger movements, although the recognition accuracy of the three models can reach over 95% with sEMG signals, the accuracy with IMU signals is too low. Only using IMU signals can’t successfully recognize finger movements. In addition, our proposed models’ recognition accuracy of arm and finger movements with one signal alone is lower than that of identifying all 20 categories of movements with the two signals together.

Thus, if the target is to recognize only arm movement, using the sEMG signal or IMU signal alone can achieve good recognition results. If the target is to recognize only finger movements, applying sEMG signals alone is also reachable to high accuracy, but applying IMU signals alone to recognition is not feasible. If the gesture recognition system aims to recognize 20-category gestures simultaneously, the two signals are recommended to be combined. The combination of sEMG and IMU signals enables the system to recognize more gestures and accomplishes better precision.

## 5. Conclusions

This work conducts a gesture recognition modeling study based on the sEMG and IMU signals. 

The conclusions drawn in the paper are as follows:

(1) A dataset containing sEMG signals and IMU signals is built through the Myo armband. The dataset includes 20 different hand gestures with a total of nearly 20,000 actions; these actions involve dynamic movements dominated by arms and static movements dominated by fingers.
(2) Based on the baseline gesture recognition models, including the two-stream CNN model, RNN model, and Transformer model, the two-stream CNN-Res model, RNN-Res model, and Transformer-CNN model are proposed, respectively. The CNN-Res model introduces the residual units and has more profound network layers; it achieves a test accuracy of 98.24% and the shortest training and test time. The RNN-Res model combines the RNN model and the CNN-Res model to enhance the degree of extracting local features, accomplishing the highest recognition accuracy. The LSTM-Res model and the GRU-Res model test accuracy are 99.67% and 99.46%, respectively. The Transformer model is incorporated with the CNN model to enhance its ability to capture local information. The modified Transformer-CNN model improves its accuracy from 71.86% to 98.96%; moreover, its shortest recognition response time of 0.0149 s for a single sample makes it highly applicable in real-time recognition and interaction systems.
(3) Through the separate recognition of arm and finger movements, the effectiveness of the combination of sEMG signals and IMU signals in the multi-category mission of this paper is proved; it turns out that simultaneously adopting two signals allows us to recognize 20 gestures and achieves the highest recognition accuracy.

Future work needs to concentrate on optimizing the parameter settings of the model. Although our proposed models achieve a high recognition precision, their training time is at the level of hours. Therefore, more research is required to establish more efficient models. In addition, deploying models in embedded systems such as real-time interfaces will also be the focus of our future research. After sorting out our dataset, we will make it publicly available on GitHub soon.

## Figures and Tables

**Table 1:** Performance results of basic models.

Model	Training Time (h)	Test Time (s)	Test Accuracy (%)
CNN	2.0	0.25	95.43
LSTM	13.3	2.12	97.10
GRU	13.8	2.45	95.91
Transformer	12.5	1.47	71.68

**Table 2:** Performance results of improved models.

Model	Training Time (h)	Test Time (s)	Single Test Time (s)	Test Accuracy (%)
CNN	2.0	0.25	0.0303	95.43
CNN-Res	4.33/1.43 (ES)	0.64	0.0343	98.24
LSTM	13.3	2.12	0.0115	97.10
LSTM-Res	18.7/8.6 (ES)	3.22	0.0312	99.67
GRU	13.8	2.45	0.0120	95.91
GRU-Res	18.9/11.7 (ES)	2.82	0.0318	99.49
Transformer	12.5	1.47	0.0126	71.68
Transformer-CNN	14.1	2.45	0.0149	98.96

**Table 3:** Accuracy results from the separate recognition.

	Models	CNN-Res		RNN-Res		Transformer-CNN	
Gestures		sEMG	IMU	sEMG	IMU	sEMG	IMU
Arm		90.16%	95.52%	98.13%	96.55%	98.13%	96.39%
Finger		95.48%	41.80%	99.12%	42.89%	98.33%	18.21%
Together		98.24%		99.67%		98.96%	
